# Integrative In Silico Identification of TP53-Associated Drug Repurposing Candidates in Lung Adenocarcinoma

**DOI:** 10.3390/ph19050761

**Published:** 2026-05-13

**Authors:** Akile Tuncal, Rasime Kalkan

**Affiliations:** 1 Department of Medical Biochemistry, Faculty of Medicine, Cyprus Health and Social Sciences University, Morphou 99750, Cyprus; 2 Department of Medical Genetics, Faculty of Medicine, European University of Lefke, Lefke 99010, Cyprus; kalkanr@yahoo.com

**Keywords:** TP53, lung adenocarcinoma, atropine, drug repurposing, in silico analysis

## Abstract

**Background/Aim:** Lung adenocarcinoma (LUAD) is the most common subtype of lung cancer and is characterized by high genetic heterogeneity and poor prognosis. TP53 is the most frequently mutated gene in LUAD and plays a critical role in tumor initiation, progression, and therapeutic resistance. The present study aimed to prioritize TP53-associated drug repurposing candidates in LUAD using an integrative in silico approach. **Materials and Methods:** A total of 1309 TP53-associated compounds were retrieved from the Gene2Drug database. Drug sensitivity profiles of lung adenocarcinoma cell lines were evaluated using PRISM Repurposing Public 22Q2 viability data obtained from the DepMap platform. Candidate compounds were ranked according to Gene2Drug significance scores (*p* < 1 × 10^−3^), and compounds with concordant sensitivity patterns in PRISM data were prioritized. **Results:** LUAD cell lines showed the strongest sensitivity to atropine (*p* = 6.83 × 10^−5^). Additionally, LUAD cell lines displayed consistent sensitivity signals for dropropizine (*p* = 8.47 × 10^−3^), terazosin (*p* = 1.11 × 10^−3^), morantel (*p* = 9.05 × 10^−3^), netilmicin (*p* = 8.37 × 10^−3^), altretamine (*p* = 9.82 × 10^−3^), and perphenazine (*p* = 9.58 × 10^−3^). These findings indicate that several non-oncology drugs exhibit distinct and reproducible sensitivity profiles in LUAD cell lines. **Conclusions**: Based on TP53-associated drug sensitivity profiles, this in silico analysis identifies atropine among the prioritized candidates, showing the strongest TP53-associated sensitivity signal in LUAD cell lines. Although experimental validation is required, the integration of independent computational datasets provides a robust framework for candidate prioritization and our findings provide a rationale for further preclinical investigation of atropine and related compounds in LUAD.

## 1. Introduction

Lung cancer is one of the most prevalent cancers worldwide and remains the leading cause of cancer-related mortality in both men and women [1]. Non-small-cell lung cancer (NSCLC) and small-cell lung cancer (SCLC) account for approximately 85% and 15% of all lung cancer cases, respectively [2]. Despite advances in treatment, clinical outcomes remain poor, with substantial early mortality and a low overall five-year survival rate [1,3]. The main histologic subtypes of NSCLC include squamous-cell carcinoma, adenocarcinoma, and large-cell carcinoma, with adenocarcinoma being the most prevalent [2,3]. Lung adenocarcinoma (LUAD), originating from glandular epithelial cells, constitutes a substantial proportion of NSCLC cases and is frequently associated with unfavorable clinical outcomes [3]. Although incidence rates have declined in recent decades, LUAD continues to represent a major global health burden due to its high mortality [4]. LUAD is characterized by an early tendency toward aggressive metastasis, most commonly affecting the brain, liver, adrenal glands, bones, and skin [5]. Because early-stage LUAD is frequently asymptomatic, diagnosis often occurs at an advanced stage, when curative treatment options are limited [5,6]. In addition, resistance to conventional chemotherapy and radiotherapy further contributes to poor prognosis [7]. Thus, there is an urgent need to identify novel therapeutic agents for LUAD, particularly for those with advanced, recurrent, or metastatic diseases.

A detailed understanding of the biological characteristics that drive LUAD initiation, progression, and treatment resistance is, therefore, essential for the development of targeted therapeutic approaches. Traditional drug discovery strategies, including cell culture- and animal-based screening models, have long been used to evaluate candidate compounds [8]. However, these protocols require a considerable amount of time and money. On the other hand, using computational methods to develop candidate drugs for LUAD is expected to significantly reduce both experimental costs and labour. Several computational methodologies have been proposed using various molecular biology databases [9]. These methods consider the drug-target network or drug clustering to identify candidate drugs. Here, we employed a computational approach to prioritize candidate compounds associated with LUAD. In this study, in silico techniques were employed to prioritize TP53-associated candidate compounds that may warrant further experimental investigation in LUAD.

The high mortality associated with LUAD is partly attributed to its complexity, particularly its large number of genetic mutations and activated pathways. The development of many genetically mutated subtypes of LUAD presents another significant challenge in the development of anti-cancer treatments. In addition to these factors, chemotherapeutics used in the treatment of LUAD often produce multidrug resistance and have severe side effects in patients. Consequently, developing therapeutics specifically effective for lung adenocarcinoma is of considerable importance. In recent years, computational methodologies have enabled the identification of candidate drugs more efficiently than experimental approaches.

**Figure 1:**
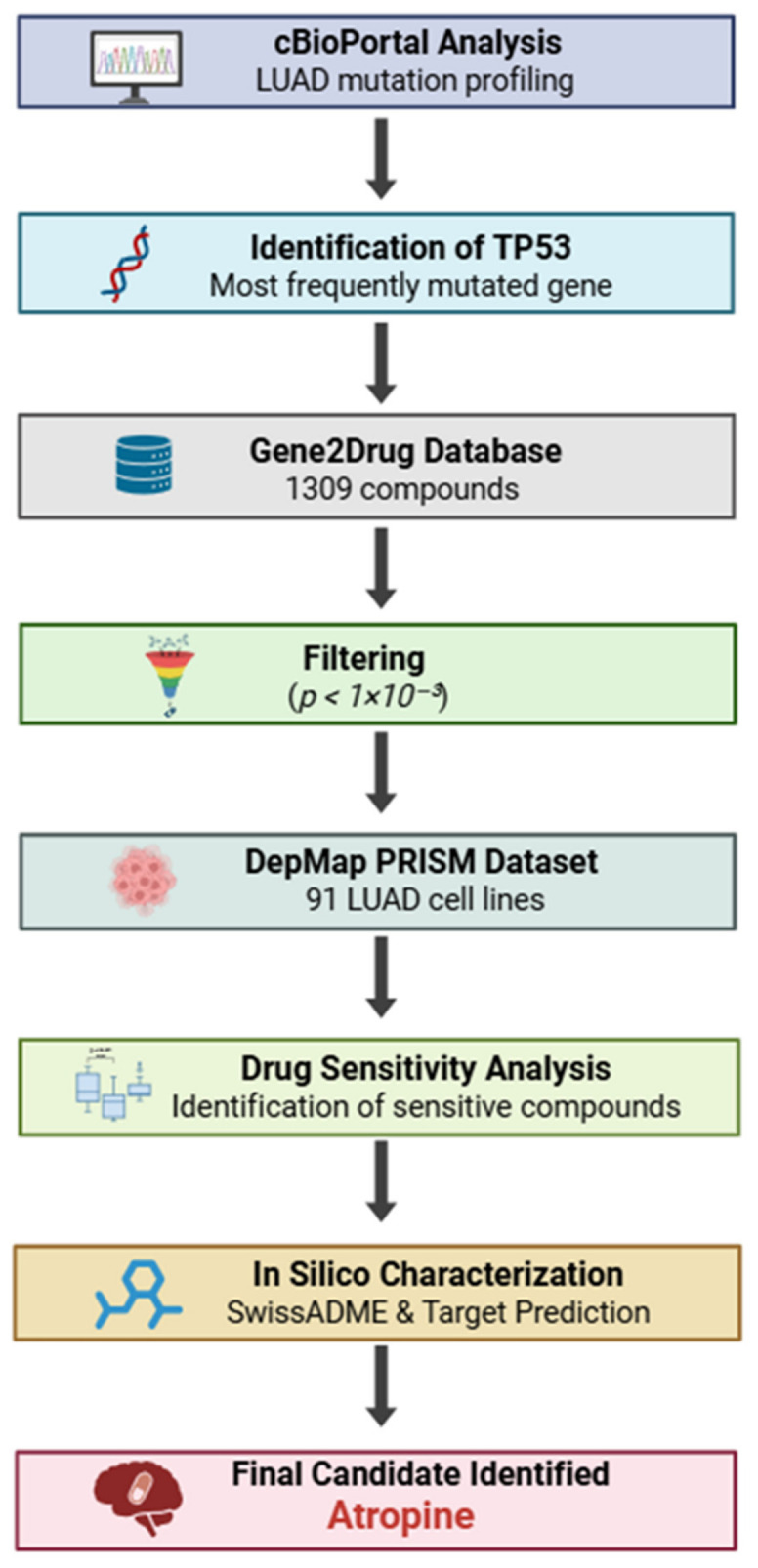
Schematic workflow of the integrative in silico analysis performed in this study. TP53-associated compounds were identified using Gene2Drug and filtered based on statistical significance. Drug sensitivity was evaluated using DepMap PRISM data from LUAD cell lines, followed by in silico characterization and target prediction analyses. This workflow led to the prioritization of atropine as a TP53-associated candidate.

The TP53 gene prevents cancer by regulating cell cycle control, DNA repair, and apoptosis [10]. Often called the guardian of the genome, it is a tumor suppressor gene and is considered protective against tumor formation. It targets cancer in multiple ways, including repairing damaged DNA by halting cell division, which may lead to apoptosis [11]. In LUAD, TP53 is frequently mutated. Significantly, high TP53 mutation rates are associated with a poor prognosis for lung cancer patients and also confer oncogenic properties to *p53* mutants. Moreover, TP53 mutations are linked to immune escape mechanisms, as they regulate PD-L1, which interacts with T cell receptors [12]. Patients with TP53 mutations may be resistant to immunotherapy. Regrettably, *p53* is difficult to target directly [13]. An alternative strategy is to focus on downstream molecular alterations and vulnerabilities associated with TP53 dysfunction. Using computational approaches to explore TP53-associated drug sensitivity patterns, the present study aims to identify candidate compounds that may support future experimental validation in LUAD, through an integrative analysis of complementary computational datasets. The overall workflow of the integrative in silico analysis performed in this study is summarized in Figure 1.

## 2. Results

**Frequently mutated genes in LUAD:** The list of the top 5 frequently mutated genes from cBioPortal is presented in Table 1. From the cBioPortal database, TP53 mutations accounted for 52.1% of genetic alterations in LUAD [14,15,16].

Gene-to-drug analysis and sensitivity of LUAD cells to candidate compounds: The list of TP53-associated compounds was produced by Gene2Drug. Compounds with Gene2Drug-derived *p* < 1 × 10^−3^ were prioritized for downstream analysis (Table 2).

**Figure 2:**
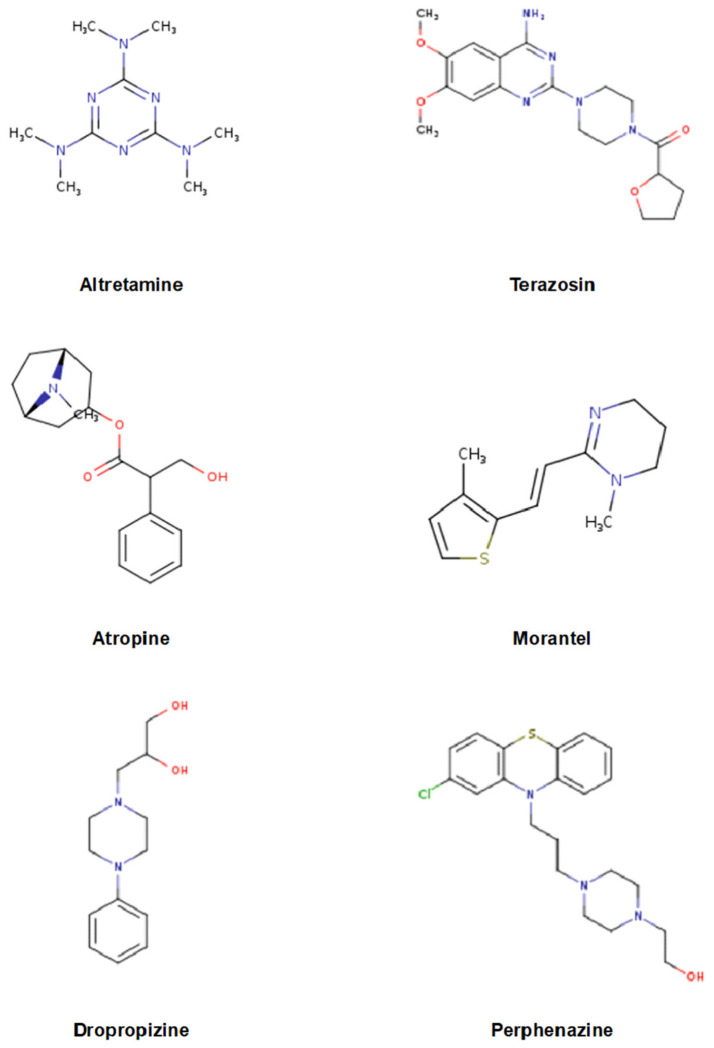
Chemical structures of the prioritized TP53-associated candidate compounds identified through the integrative in silico workflow. The compounds shown (dropropizine, terazosin, morantel, atropine, altretamine, and perphenazine) correspond to those listed in Table 2 and were selected based on Gene2Drug prioritization and consistent drug sensitivity patterns observed in DepMap PRISM LUAD cell line data.

Drug sensitivity was evaluated using PRISM Repurposing Public 22Q2 data from DepMap [17,18,19], and all 1309 compounds were assessed across LUAD cell lines. Based on Gene2Drug prioritization (*p* < 1 × 10^−3^) and consistent sensitivity signals in PRISM viability data, candidate compounds were selected for further in silico characterization. After drug-likeness/ADME filtering using SwissADME, netilmicin was excluded, yielding six prioritized TP53-associated compounds, whose chemical structures are shown in Figure 2. Among these, atropine showed the strongest sensitivity signal (*p* < 0.001) (Table 2). In addition, dropropizine, terazosin, morantel, netilmicin, altretamine, and perphenazine demonstrated statistically significant and consistent sensitivity signals in LUAD cell lines (*p* ≤ 0.01) (Table 2). These findings indicate consistent sensitivity patterns across datasets, supporting the robustness of the candidate prioritization strategy.

**Figure 3:**
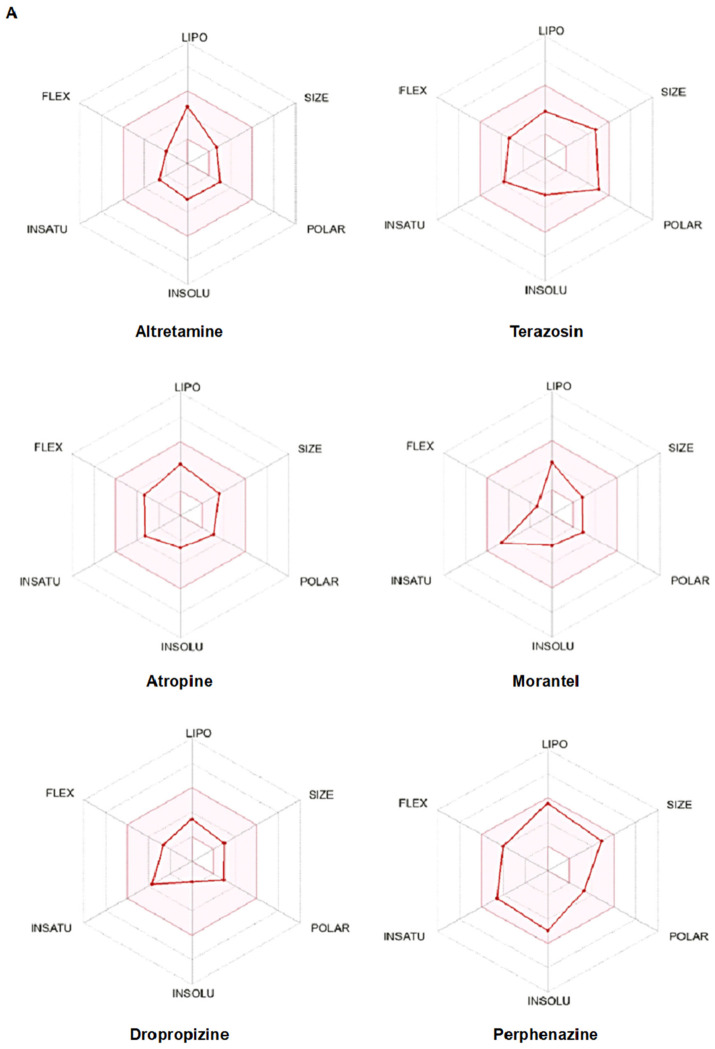

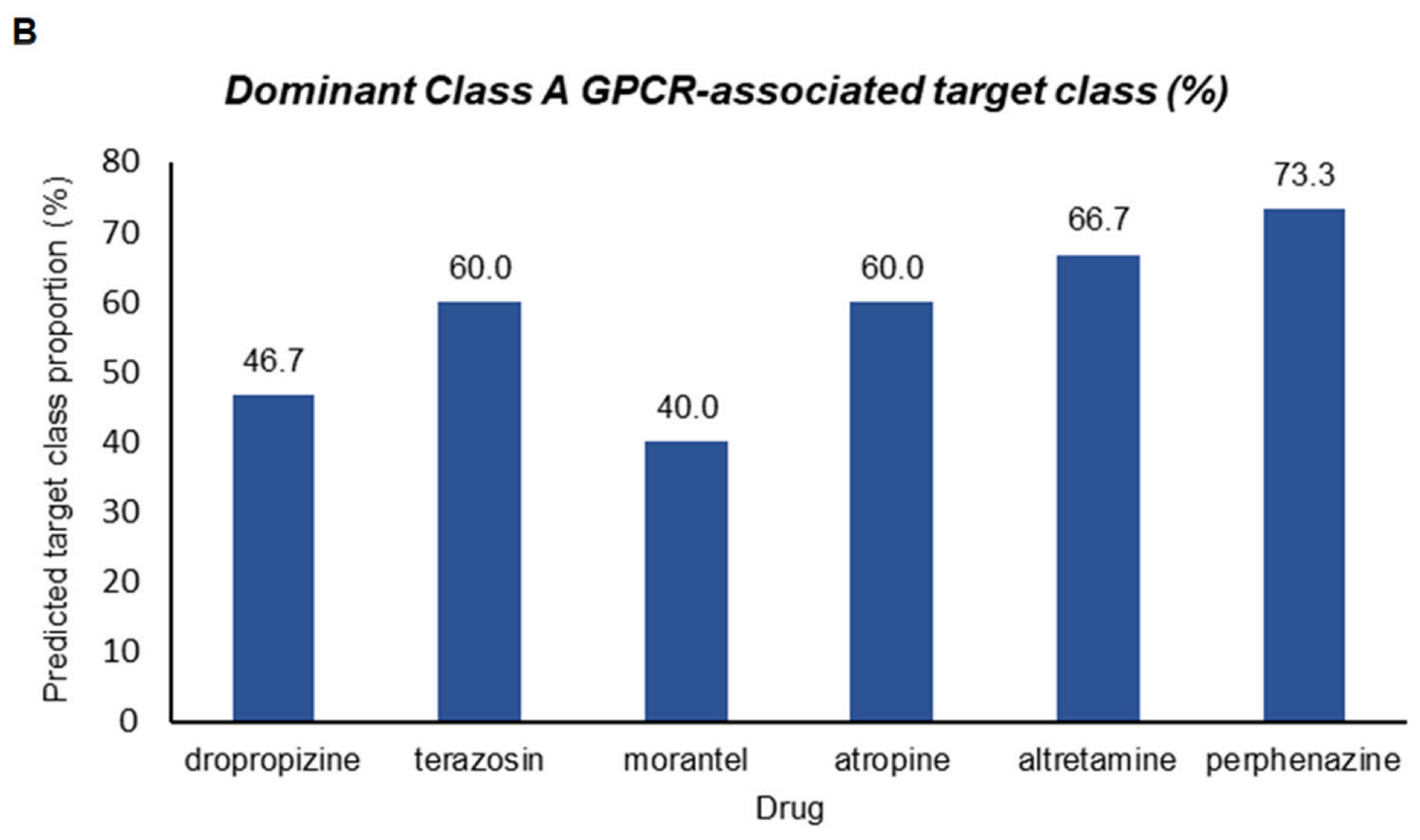
In silico characterization of prioritized TP53-associated candidate compounds. (**A**) SwissADME bioavailability radar plots for dropropizine, terazosin, morantel, atropine, altretamine, and perphenazine. The pink area indicates the optimal physicochemical space for oral bioavailability. (**B**) Dominant predicted target class distribution of prioritized TP53-associated candidate compounds based on SwissTargetPrediction analysis. Bars represent the proportion of predicted Class A G protein-coupled receptor (GPCR)-associated targets for each compound.

**In silico analysis of compounds:** The bioavailability radars provide a pharmacological representation of the selected compounds. Within the graphs, the area highlighted in pink represents the optimal ranges for each physicochemical property (lipophilicity, size, polarity, solubility, saturation, and flexibility) relevant to oral bioavailability and systemic exposure [20] (Figure 3A). We utilized the web tool SwissTargetPrediction to identify alternative targets for compounds [21]. We obtained high-probability target predictions for atropine, dropropizine, terazosin, morantel, altretamine, and perphenazine (Figure 3B). Accordingly, netilmicin was not included in subsequent analyses due to not meeting drug-likeness/ADME criteria in SwissADME [20]. Reverse-screening approaches for mapping compound–target interactions have been described previously [22]. Notably, Class A GPCRs represented the dominant predicted target family across the prioritized compounds, indicating a shared pharmacological feature among TP53-associated candidates (Figure 3B).

The physicochemical properties of the prioritized compounds, including lipophilicity (XlogP3), topological polar surface area (TPSA), hydrogen bonding capacity, and molecular flexibility, were evaluated to assess drug-likeness. Most compounds were found to fall within acceptable ranges, suggesting favorable pharmacokinetic properties such as membrane permeability and oral bioavailability. These descriptors are critical in predicting drug-like behavior and further support the suitability of the identified candidates for drug development, as summarized in Table 3 [20,23].

**Figure 4:**
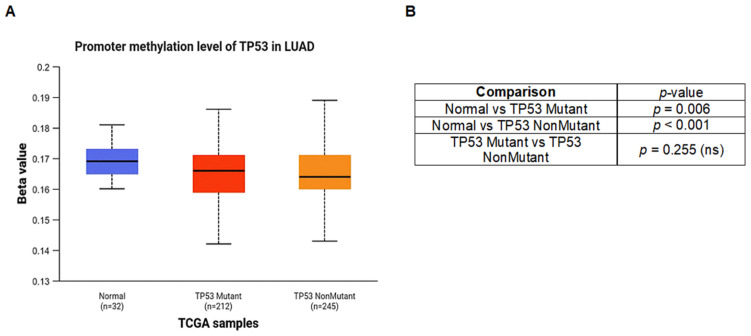
TP53 promoter methylation profile based on TP53 mutation type. (**A**) Distribution of TP53 promoter methylation (β values) in normal lung samples, TP53-mutant LUAD, and TP53-nonmutant LUAD samples derived from TCGA. (**B**) Summary of statistical comparisons of TP53 promoter methylation levels among the indicated groups. Statistical significance was assessed using the statistical test implemented by the TCGA-based analysis platform. A *p*-value < 0.05 was considered statistically significant (ns: not significant). In the box plots, the median is represented by the central line, the interquartile range (IQR) is represented by the box, and the whiskers indicate the minimum and maximum values.

**TP53 promoter methylation analysis:** TP53 promoter methylation patterns were examined in LUAD samples according to TP53 mutation status and compared with normal lung tissues. As shown in Figure 4A, promoter methylation levels were lower in LUAD samples than in normal tissues. This decrease was statistically significant in both TP53-mutant (*p* = 0.006) and TP53-wild-type LUAD samples (*p* < 0.001) when compared with normal lung tissues (Figure 4B). In contrast, no statistically significant difference in promoter methylation was observed between TP53-mutant and TP53-wild-type tumors (*p* = 0.255). In LUAD samples, the β values fell within hypomethylation ranges previously reported in the literature [24,25] (Figure 4A). Together, these findings indicate that reduced TP53 promoter methylation is a common epigenetic characteristic of LUAD and appears to be largely independent of mutation status.

**Figure 5:**
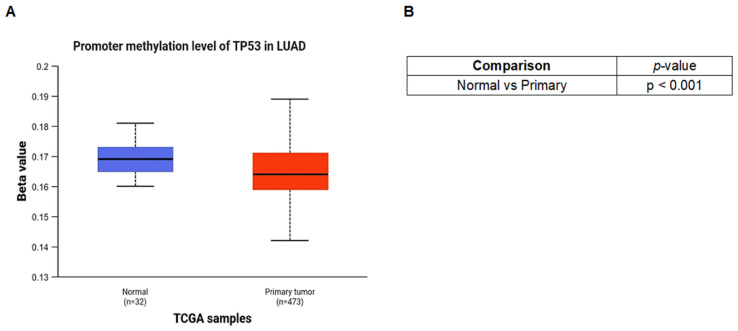
TP53 promoter methylation profile based on sample type. (**A**) Distribution of TP53 promoter methylation (β values) in normal lung and primary LUAD samples from TCGA. (**B**) Statistical comparison of TP53 promoter methylation levels between normal and primary tumor samples. Statistical significance was assessed using the statistical test implemented by the TCGA-based analysis platform. A *p*-value < 0.05 was considered statistically significant. In the box plots, the median is represented by the central line, the interquartile range (IQR) is represented by the box, and the whiskers indicate the minimum and maximum values.

To further explore this pattern, TP53 promoter methylation levels were also compared based on sample type. Primary LUAD tumors exhibited significantly lower promoter methylation levels than normal lung tissues (Figure 5A). This difference was confirmed by statistical analysis (*p* < 0.001; Figure 5B), supporting the observation that TP53 promoter hypomethylation is a characteristic alteration in primary LUAD.

## 3. Discussion

There is a critical need to develop novel therapeutic strategies for LUAD that incorporate biologically informed approaches. LUAD poses a considerable challenge within the field of oncology, primarily due to its high mortality rate, prevalent genetic mutations, and resistance to standard therapeutic approaches [26,27]. This study used computational methods to identify candidate compounds associated with TP53, given that TP53 is one of the most frequently mutated genes in LUAD. In silico methodologies, such as Gene2Drug and DepMap, identified atropine as a candidate compound with a notable sensitivity profile in LUAD cell lines. The findings of this study provide a computational rationale for further investigation and highlight the growing importance of in silico approaches in contemporary drug discovery. Rather than aiming to establish direct therapeutic efficacy, this work emphasizes the value of genomics-guided prioritization strategies in genetically complex malignancies such as LUAD.

Referred to as the “guardian of the genome,” TP53 is an essential tumor suppressor gene associated with numerous cancer-related pathways, including the regulation of the cell cycle, DNA repair, and the process of apoptosis [28]. In LUAD, TP53 is among the most frequently mutated genes, and its alteration has been closely associated with tumorigenesis, treatment resistance, and poor clinical outcomes. Accumulating evidence increasingly points to a role for TP53 mutations in immune evasion, partly through their influence on PD-L1 regulation, a mechanism that may limit the effectiveness of immunotherapies across several cancer types [29,30]. Although TP53 remains a cornerstone of tumor biology, efforts to target it directly at the pharmacological level have been largely unsuccessful, mainly because of its structural complexity and multifunctional nature [31]. As a result, attention has gradually shifted toward identifying downstream molecular weaknesses that arise from TP53 dysfunction. Within this framework, the prioritization of atropine as a TP53-associated candidate in the present study highlights the promise of indirect, pathway-oriented therapeutic strategies.

In addition to genetic alterations, epigenetic mechanisms may further contribute to TP53 dysregulation in LUAD. Consistent TP53 promoter hypomethylation was observed in LUAD samples when compared with normal lung tissues, irrespective of TP53 mutation status (Figure 4A and Figure 5A). This observation indicates that altered promoter methylation represents a broader epigenetic characteristic of LUAD rather than a mutation-dependent phenomenon. Importantly, these results should be interpreted as reflecting changes in the regulatory context, rather than evidence of functional TP53 reactivation.

The relationship between TP53 promoter hypomethylation and atropine sensitivity warrants further investigation. While our analysis demonstrates consistent TP53 promoter hypomethylation in LUAD (Figure 4A), the correlation between this epigenetic alteration and the observed atropine sensitivity remains unexplored. TP53 promoter hypomethylation may influence chromatin accessibility and transcriptional dynamics, potentially modulating cellular vulnerabilities that contribute to drug sensitivity patterns. Future experimental studies should examine whether LUAD cell lines with stronger atropine sensitivity exhibit more pronounced TP53 promoter hypomethylation, as such correlations could provide mechanistic insights into epigenetic determinants of therapeutic response and support biomarker development for TP53-targeted approaches in LUAD.

Traditional drug discovery approaches are often time-consuming, costly, and ethically challenging, particularly during preclinical stages that rely on animal experimentation. In this context, in silico screening and simulation-based strategies have become valuable tools for prioritizing large compound libraries prior to resource-intensive experimental testing [32]. In the present study, TP53-associated candidate compounds were identified using the Gene2Drug database, while drug sensitivity profiles in LUAD cell lines were examined using PRISM Repurposing data from the DepMap platform. The integration of these computational resources enabled high-throughput assessment of 1309 compounds and facilitated the prioritization of candidates based on TP53-associated sensitivity patterns, with atropine exhibiting the strongest signal (*p* = 6.83 × 10^−5^). This integrative approach, combining complementary computational datasets, provides cross-dataset support and enhances the robustness of the candidate prioritization despite the absence of experimental validation.

Atropine is a tropane alkaloid that is well established in clinical practice due to its anticholinergic properties and broad use across multiple medical indications [33]. Notably, atropine is routinely administered for the management of acute cholinergic syndrome induced by irinotecan-based chemotherapy regimens [31]. Premedication with atropine before irinotecan treatment in metastatic colorectal cancer patients plays a key role in preventing early-onset diarrhoea [34]. Mirzaei et al. [35] demonstrated the antimicrobial and anti-tumor relevance of atropine, although further studies are needed to clarify these effects in cancer. However, studies on atropine’s direct anticancer effects are limited, and there is a lack of sufficient data on its cytotoxicity against cancer cells or its ability to inhibit tumor progression [35]. More recently, Lau et al. [36] proposed that atropine may influence apoptosis and cell proliferation pathways, including EGFR/ERK signaling, which could partly explain the sensitivity signals observed in LUAD cell lines. In line with this notion, Berne et al. demonstrated in a preclinical model that a cholinergic antagonist (APS8) induced apoptosis and delayed tumor growth in LUAD cells characterized by high expression of α7 nicotinic acetylcholine receptors (α7nAChR) [37]. Although APS8 is not atropine, these findings support the broader relevance of cholinergic signaling in LUAD biology.

Taken together, these observations suggest that atropine may affect tumor-related pathways in LUAD, potentially through modulation of α7nAChR-associated mechanisms, and help to explain its prioritization in the present analysis. While GPCR-associated target classes were prevalent among several prioritized compounds, the consistent enrichment observed for atropine points to cholinergic signalling as a biologically plausible context in TP53-altered LUAD. Importantly, this does not imply direct therapeutic efficacy. Instead, these findings point to pathway-level vulnerabilities that may warrant further experimental exploration. Accordingly, any consideration of atropine as a candidate for oncological repurposing will require additional studies to clarify its underlying mechanisms, define appropriate dosing ranges, and assess its potential use alongside established therapeutic strategies. However, given its known side-effect profile, careful consideration of dosing and safety would be required if atropine were to be explored for anti-tumor applications. Since atropine is known to cause side effects like increased heart rate and nervous system disturbances, and its potential use in cancer treatment would require careful dose adjustment and close patient monitoring to ensure safety [38].

This study highlights the potential of atropine, but it has some limitations that must be addressed. Furthermore, reliance on computational methods may overlook potential pharmacokinetic and pharmacodynamic challenges that are not captured in current datasets. Differences in absorption, distribution, and metabolic handling of atropine may influence its biological activity in oncological settings. To gain a better understanding, detailed pharmacokinetic studies should be conducted in preclinical research [39].

While the present computational analysis identifies atropine as a promising TP53-associated candidate for LUAD, clinical translation would likely benefit from targeted delivery strategies to enhance therapeutic specificity and minimize systemic adverse effects. The loading of atropine onto nanocarriers represents a promising approach to achieve tumor-selective delivery while preserving normal tissues. Several nanocarrier systems, including lipid nanoparticles, polymeric micelles, and targeted drug delivery vehicles equipped with tumor-specific ligands (such as EGFR or folate receptor targeting moieties), could potentially enhance the therapeutic index of atropine in LUAD [40,41]. Such targeted delivery mechanisms could address the inherent limitation of atropine’s systemic toxicity profile, including cardiovascular and neurological side effects, by concentrating drug accumulation within tumor tissue while reducing exposure to normal organs [42]. Additionally, nanoformulations may offer advantages in pharmacokinetic optimization, controlled release kinetics, and enhanced bioavailability [43]. Future preclinical studies should prioritize the development and evaluation of targeted atropine nanoformulations alongside conventional formulations to establish the optimal delivery strategy for potential clinical applications in LUAD. Taken together, these considerations underscore the need for experimental validation and, ultimately, clinical studies to assess the relevance of the present computational findings. Therefore, further in vitro and in vivo studies are required to specifically validate the therapeutic relevance of atropine in LUAD.

Furthermore, the study focused only on LUAD cell lines, and its applicability to other cancer subtypes is uncertain. Some early studies using breast and colorectal cancer models hint that atropine might work against more than one type of tumor [35]. This suggests that exploring its effects across different cancers could be beneficial. Additionally, atropine’s safety and tolerability profile, particularly in oncological settings such as chemotherapy premedication, appears acceptable based on existing clinical data [36]. Nevertheless, thorough evaluation of off-target effects and dose optimization remains critical if atropine is to be explored for anti-tumor applications [38].

Future research should include preclinical studies to confirm the anti-tumor relevance of atropine, investigate its molecular mechanisms, and evaluate its safety and efficacy in vivo. Ahmed et al. [44] reported that atropine suppresses epithelial–mesenchymal transition and reduces stemness in drug-resistant breast cancer cells, highlighting its potential anti-tumor relevance beyond supportive care. This points to atropine having potential anti-cancer effects beyond its usual supportive uses. Building on these observations, future work may explore whether atropine exerts additive or synergistic effects when administered alongside immune checkpoint inhibitors or targeted therapies. Investigating such combination-based approaches could help define the potential positioning of atropine within broader therapeutic strategies for LUAD [45,46].

In clinical practice, atropine is widely used to manage chemotherapy-associated adverse effects [47]. However, its role as a direct anticancer agent in LUAD remains uncertain and requires careful investigation [45,46]. Overall, the present findings indicate that atropine, among several TP53-associated candidates, warrants further investigation in LUAD. Given its diverse biological activities, subsequent experimental studies in TP53-mutant LUAD models should focus on dose–response relationships, cholinergic signalling-related pathways, and rational combination strategies with current LUAD treatments.

## 4. Materials and Methods

**Data sources and computational tools:** The cBio Cancer Genomics Portal (cBioPortal) database (https://www.cbioportal.org/, accessed on 20 May 2025) explores and visualizes a large amount of genomic data from patients with cancer, sourced from the Cancer Genome Atlas (TCGA) [14]. We used cBioPortal to analyse the top 5 mutated genes in LUAD [14,15,16]. An overview of the integrative in silico workflow applied in this study is presented in Figure 1.

We utilized in silico screening tools to identify TP53-associated candidate compounds [17,18,19]. For this purpose, we used the Gene2Drug database (https://gene2drug.tigem.it/, accessed on 22 May 2025), which ranks 1309 compound molecules associated with TP53-related biological pathways [48]. To evaluate drug sensitivity profiles of these compounds, we analyzed data from the DepMap platform (https://depmap.org/portal/, accessed on 26 May 2025), specifically the PRISM Repurposing Public 22Q2 dataset, which includes viability assay results from 91 LUAD cell lines, which was used as an independent dataset to support candidate prioritization. This dataset was subsequently analyzed to assess the relative sensitivity of LUAD cell lines to TP53-associated candidate compounds [17,18,19]. The complete list of analyzed TP53-associated compounds along with their corresponding PRISM sensitivity profiles is provided in Supplementary Table S1.

**Analysis of gene-to-drug interactions:** The Gene2Drug database identified candidate compounds associated with TP53-related molecular functions [48]. Compounds were ranked by Gene2Drug significance, and those with *p* < 1 × 10^−3^ were considered relevant.

**In silico analysis of candidate compounds:** SwissADME (http://www.swissadme.ch/, accessed on 15 April 2025) was utilized for an in silico analysis of compounds. This complementary online software application facilitates the assessment of pharmacokinetics, drug-likeness (the probability of oral bioavailability), and medicinal chemistry friendliness (PAINS) of small molecules [20]. Furthermore, target prediction was performed using the SwissTargetPrediction web tool [21].

**Promoter methylation analysis of TP53:** DNA methylation and gene expression data were retrieved from The Cancer Genome Atlas (TCGA) database. TP53 mutation status was obtained from TCGA whole-exome sequencing data, and Mutation Annotation Format (MAF) files derived from VarScan2 were downloaded from the Genomic Data Commons portal. Samples with and without TP53 mutations were matched with corresponding RNA-seq data [49,50].

Promoter methylation and TP53 expression levels were compared between TP53-mutant and TP53-wild-type LUAD samples using publicly available TCGA-based analysis resources [49,50]. Statistical comparisons between groups were performed using the test implemented by the respective TCGA-based analysis platform, and the *platform-reported p*-values were used for downstream interpretation.

## 5. Conclusions

In the present study, we identified and prioritized TP53-associated candidate compounds for LUAD using computational methods. Our results highlight atropine as a prioritized candidate based on TP53-associated drug sensitivity profiles, rather than as a confirmed therapeutic agent. While targeting p53 in cancer treatment remains a promising research avenue, the complexity of its biological functions and the heterogeneity of TP53 mutations continue to present significant challenges.

Although atropine is primarily recognized as a muscarinic receptor antagonist, its potential relevance in cancer biology may be linked to the broader interplay between cholinergic signaling and tumor-related pathways. Within this context, the present in silico analysis provides a biologically informed rationale for further experimental investigation and contributes to the growing body of research on computational drug repurposing in LUAD. Importantly, beyond the identification of individual compounds, this study presents a structured and integrative in silico approach that links TP53 mutation status with drug sensitivity and target prediction data. This framework offers a reproducible strategy for candidate prioritization in LUAD. Nevertheless, additional experimental studies will be required to determine the biological relevance and translational potential of atropine and other prioritized compounds.

## Figures and Tables

**Table 1:** Top five most frequently mutated genes in LUAD (cBioPortal).

Gene	Mutation Count	Number of Samples	Frequency
*TP53*	314	295	52.1%
*TTN*	679	272	48.1%
*MUC16*	424	242	42.8%
*CSMD3*	394	226	39.9%
*RYR2*	395	217	38.3%

**Table 2:** TP53-associated compounds identified by Gene2Drug and their sensitivity in LUAD cell lines.

Compound Name	Pearson	Spearman	Slope	Intercept	*p*-Value (Linregress)
**dropropizine**	0.372	0.265	1.59 × 10^−1^	−1.99 × 10^−2^	8.47 × 10^−3^
**terazosin**	0.452	0.441	2.18 × 10^−1^	−5.01 × 10^−1^	1.11 × 10^−3^
**morantel**	−0.359	−0.414	−1.85 × 10^−1^	3.02 × 10^−1^	9.05 × 10^−3^
**netilmicin**	−0.373	−0.368	−9.00 × 10^−2^	2.63 × 10^−1^	8.37 × 10^−3^
**atropine**	**0.537**	**0.557**	**2.46 × 10^−1^**	−**9.56 × 10^−2^**	**6.83 × 10^−5^**
**altretamine**	0.365	0.382	1.56 × 10^−1^	1.62 × 10^−1^	9.82 × 10^−3^
**perphenazine**	−0.367	−0.446	−1.21 × 10^−1^	1.52 × 10^−1^	9.58 × 10^−3^

Bold formatting indicates prioritized candidate compounds and the most significant sensitivity-associated parameters.

**Table 3:** Drug-likeness properties of prioritized compounds based on SwissADME analysis. Physicochemical descriptors were calculated using SwissADME and evaluated according to established drug-likeness criteria [20,23].

	Dropropizine	Terazosin	Morantel	Atropine	Altretamine	Perphenazine	Optimal Range
**Lipophilicity (*XlogP3*)**	0.55	1.26	1.93	**1.83**	2.73	4.20	−0.7 to +5.0
**Molecular weight (g/mol)**	236.31	387.43	220.33	**289.37**	210.28	403.97	150–500
**TPSA (Å^2^)**	46.94	103.04	43.84	**49.77**	48.39	55.25	20–130
**Solubility (*log S, ESOL*)**	−1.65	−2.97	−2.54	**−2.67**	−2.96	−4.92	≤6
**Fraction Csp^3^ (*Saturation*)**	0.54	0.53	0.42	**0.59**	0.67	0.43	≥0.25
**Rotatable bonds (*Flexibility*)**	4	5	2	**5**	3	6	≤9
**H-bond acceptors**	3	6	1	**4**	3	3	≤10
**H-bond donors**	2	1	0	**1**	0	1	≤5

**Abbreviations:** XlogP3, predicted lipophilicity (log P); TPSA, topological polar surface area; ESOL, estimated solubility; Csp^3^, fraction of sp^3^-hybridized carbons. Bold formatting indicates emphasized physicochemical descriptors and the most favorable drug-likeness parameters associated with atropine.

## Data Availability

The original contributions presented in this study are included in the article/Supplementary Materials. The data used in this study are available in the Gene2Drug and DepMap databases. Further inquiries can be directed to the corresponding author(s).

## Supplementary Materials

The following supporting information can be downloaded at: https://www.mdpi.com/article/10.3390/ph19050761/s1, Table S1: Gene Ontology Biological Process (GO_BP), Cellular Component (GO_CC), Molecular Function (GO_MF), and merged Gene2Drug-derived TP53-associated compound datasets with corresponding PRISM sensitivity data.
